# Surgical intervention of Lemierre’s syndrome: a case report and review of the literature

**DOI:** 10.1186/s13256-024-04584-2

**Published:** 2024-05-31

**Authors:** Yiqi Pan, Zhihong Shi, Bin Ye, Qian Da, Chaofu Wang, Yilin Shen, Mingliang Xiang

**Affiliations:** 1 Department of Otolaryngology and Head and Neck Surgery, Ruijin Hospital, Shanghai Jiao Tong University School of Medicine, Shanghai, China; 2 Department of Pathology, Ruijin Hospital, Shanghai Jiao Tong University School of Medicine, Shanghai, China

**Keywords:** Case report, Head and neck infection, Internal jugular vein thrombosis, Lemierre’s syndrome, Surgical treatment

## Abstract

**Background:**

Lemierre’s syndrome is a fatal and rare disease that is typically characterized by oropharyngeal infection and internal jugular vein thrombosis. Timely institution of appropriate antibiotics is the standard treatment.

**Case presentation:**

The authors report a case of Lemierre’s syndrome. A 67-year-old male patient of Han ethnicity in China suffered from a large inflammatory neck mass involving left internal jugular vein thrombosis diagnosed as Lemierre’s syndrome and finally cured by surgical treatment. In addition, a literature review was carried out through PubMed using the terms “Lemierre’s syndrome/disease and review, meta-analysis or retrospective study” and “Lemierre’s syndrome/disease and internal jugular vein”. This search yielded six articles that recorded surgical methods such as drainage, craniotomy, tooth extraction, and ligation of the occluded vein to give clinicians more ideas about the treatment of the Lemierre’s syndrome.

**Conclusion:**

This is the first review to summarize the conditions under which surgical treatment are conducted. Additionally, this is the first report of such a large inflammatory neck mass that was completely cured by surgical resection and internal jugular vein ligation. The authors also offer several conclusions regarding surgical intervention in Lemierre’s syndrome for the first time.

## Background

Internal jugular vein (IJV) thrombosis is a relatively rare and urgent disease. In a retrospective study, the number of such cases occurring from 2001 to 2008 was 2.5 times that occurring from 1991 to 2000 and 20 times that occurring from 1980 to 1990 [1]. The reasons behind the increasing incidence of IJV thrombosis include the increase in antibiotic resistance, widespread use of hemodialysis, general application of central venous catheters, expansion of assisted reproductive technology, and increasing incidence of cancer [2]. In a 9-year retrospective study of 1948 patients with deep vein thrombosis, only 29 patients developed IJV thrombosis, of whom 23 had IJV thrombosis secondary to another condition, such as a malignancy (for example, Trousseau syndrome), central venous catheter implantation, or ovarian hyperstimulation syndrome (OHSS) [3]. In addition, bilateral internal jugular vein thrombosis is an important indicator of malignant tumors. In a 5-year retrospective study of 41 patients with IJV thrombosis in Germany, paraneoplastic thrombosis accounted for 54% of cases; of these cases, otolaryngology head and neck diseases accounted for 68%. The other patients mostly had inflammatory diseases [4].

In the ear, nose, and throat (ENT) field, IJV thrombosis is commonly associated with Lemierre’s syndrome (LS), which is a complication of infectious diseases, such as otitis media and oropharyngeal abscess or infection. LS is commonly defined by the following diagnostic criteria: (1) oropharyngeal infection; (2) internal jugular vein thrombophlebitis or thrombosis; (3) septic emboli at a remote site, more frequently the lungs; and (4) isolation of Fusobacterium nucleatum on blood culture [5]. LS is usually accompanied by septic emboli in the lungs or other organs [6]. Under some rare conditions, LS can also be triggered by tooth extraction [7]. Pulmonary embolism, with an incidence of approximately 10%, and postthrombotic syndromes, such as limb pain, heaviness, venous dilatation, edema, pigmentation, nutritional skin changes, and venous ulcers, are complications of IJV thrombosis [4]. Therefore, ENT doctors should give enough attention to patients with IJV thrombosis to avoid the disastrous results caused by pulmonary or cerebral thrombosis.

To the best of our knowledge, this is the first case of such a large infectious neck mass with internal jugular vein thrombosis that was completely cured by surgical intervention.

## Case presentation

**Fig. 1:**
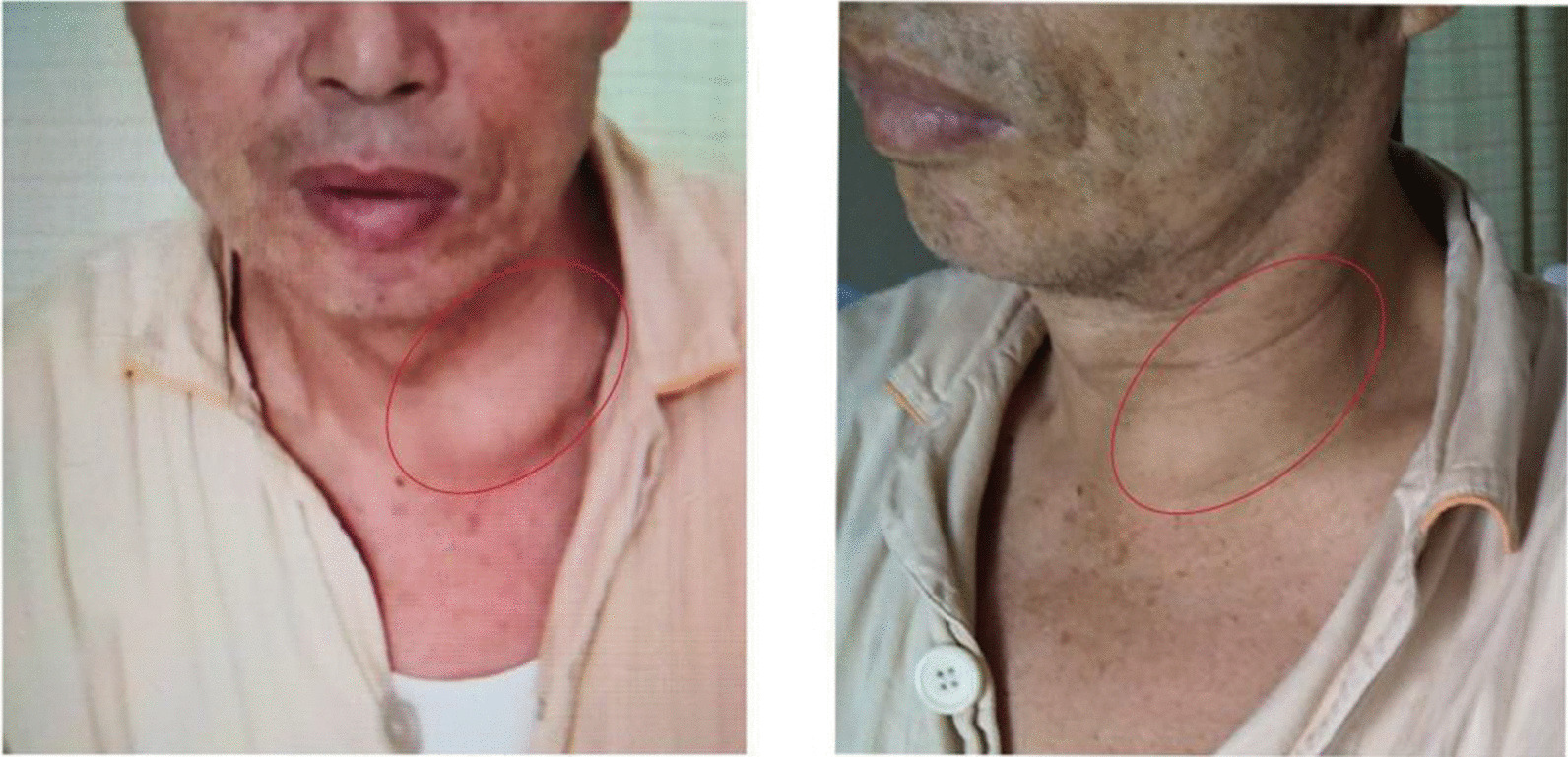
Picture of the patient’s neck showed a huge mass with tenderness (red circles)

**Fig. 2:**
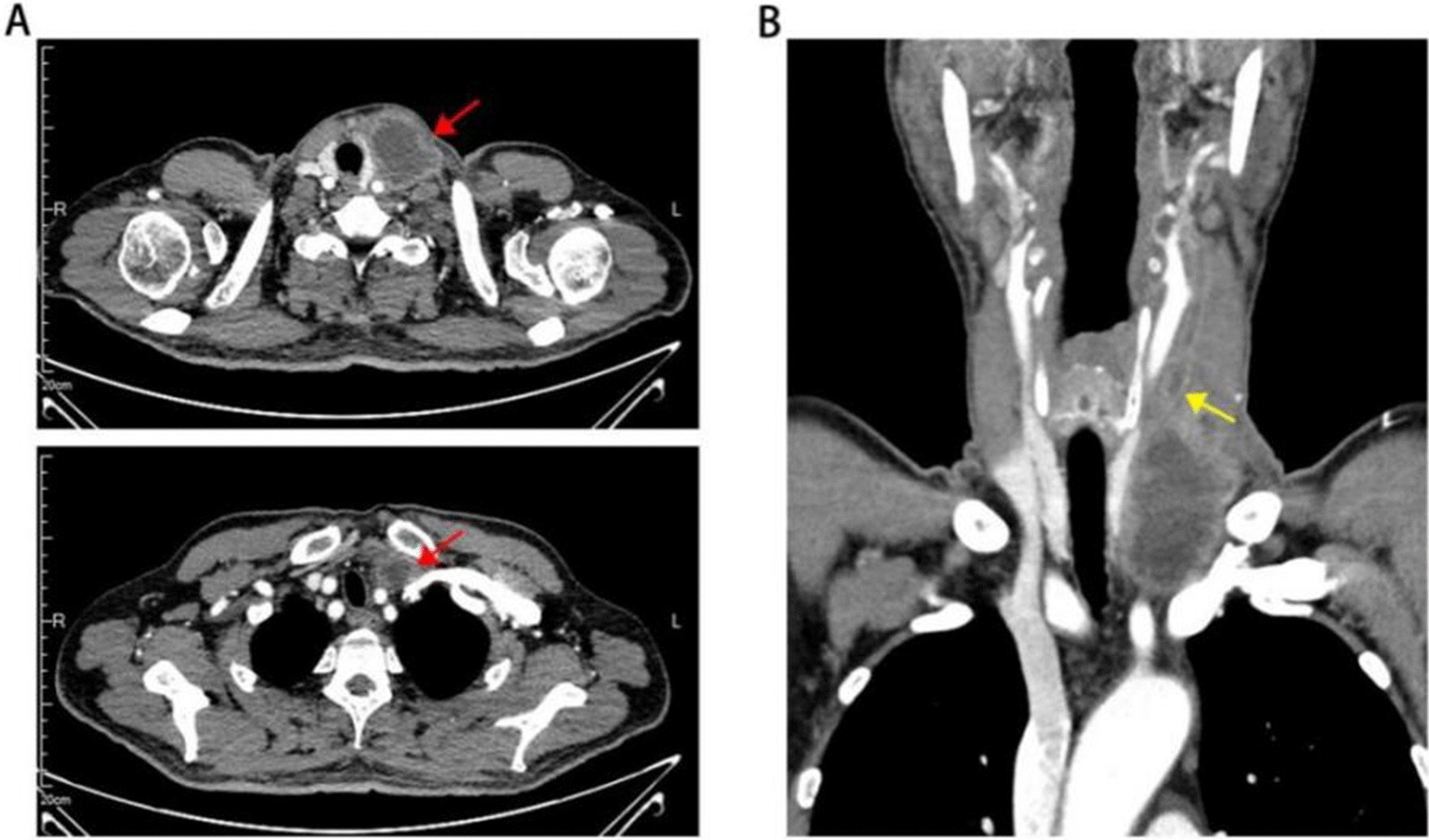
**A** Coronal plane of computed tomography showed neck mass spread down to superior mediastinum with iquefactive necrosis in the center (red arrows). Trachea was oppressed to the right side. **B** Significant intraluminal filling defect and thrombosis was found in internal jugular vein (yellow arrow)

A 67-year-old Chinese male of Han ethnicity developed pain in the left neck 14 days prior after eating mud fish. He was healthy and denied a history of infectious diseases, chronic diseases, thrombotic disease, surgical trauma, blood transfusion, allergies, or contact with poisonous substances. Physical examination on admission revealed the following: fever, chills, fatigue, mild dysuria, diffusive swelling pain of the neck on the left side, and high skin temperature. His left neck was tender and edematous with cellulitis. The mass was scleroid with a liquefied center. Other parameters were as follows: white blood cell (WBC) count, 12.2 × 10^9^/L; neutrophil count, 11.76 × 10^9^/L; platelet (PLT) count, 51 × 10^9^/L; C-reactive protein (CRP) level, 146 mg/L; procalcitonin (PCT), 156.99 ng/mL; temperature, 39.5 °C; respiratory rate, 24 breaths per minute; pulse, 118 beats per minute; and blood pressure (BP), 109/61 mmHg. The patient stated that he had been to many hospitals in the last 2 weeks and that the use of antibiotics such as ceftriaxone and metronidazole slightly alleviated his neck pain at first. However, the effect was temporary and no longer present after he transferred to Shanghai, and 3 days before he presented to our hospital, he noticed extreme swelling and felt increased pain in the area of his neck mass (Fig. 1). A series of further examinations were performed, with the following results: glucose, 22.13 mmol/L; activated partial thromboplastin time (APTT), 43.1 seconds; prothrombin time (PT), 17.3 seconds; fibrinogen (Fg), 5.0 g/L; fibrin/fibrinogen degradation products (FDP), 7.3 mg/L; and D-dimer (D-D), 2.1 mg/L. Urine analysis was positive for glucose, blood, protein, and white blood cells. Infectious diseases and acute nephrology were considered. The culture of blood and fluid obtained from the mass was negative for any bacteria including anaerobic bacteria, mycoplasma, or fungus. Ultrasound revealed mixed echogenicity in the left neck mass that was irregular in shape. The mass was approximately 74 × 37 mm in size. Color Doppler revealed generalized thrombosis of the internal jugular vein. CT of the chest and neck was conducted and suggested that the cervical abscess extended to the thorax and superior mediastinum, without a signal from the left internal jugular vein. Multiple enlarged cervical lymph nodes were observed. The trachea and left thyroid were also compressed (Fig. 2). Video laryngoscopy excluded the possibility of pyriform sinus fistula or any foreign body. Pus obtained from the mass showed many neutrophils and large amounts of necrotic tissue, and 14 days of combination antibiotic treatment (imipenem and teicoplanin) and regular insulin therapy in our hospital returned the patient’s temperature, routine blood markers, CRP and PCT levels, and coagulation function to normal. However, the neck mass remained. Therefore, the surgery department was consulted.

**Fig. 3:**
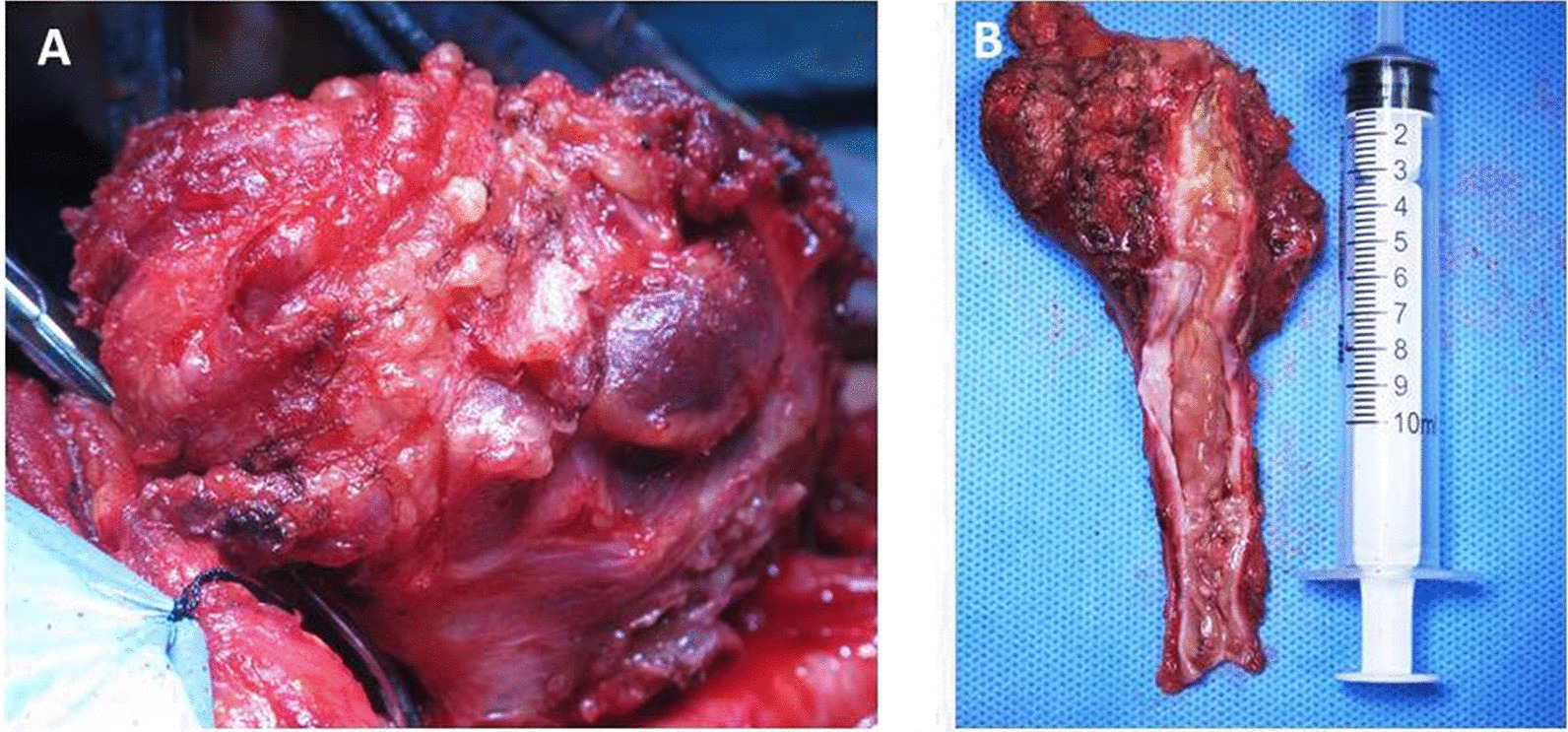
Intraoperative view showed ligation of internal jugular vein and separation of the mass (**A**). The resected neck mass as well as left internal jugular vein was shown. Intraluminal thrombosis could be seen clearly when opening the internal jugular vein (**B**)

**Fig. 4:**
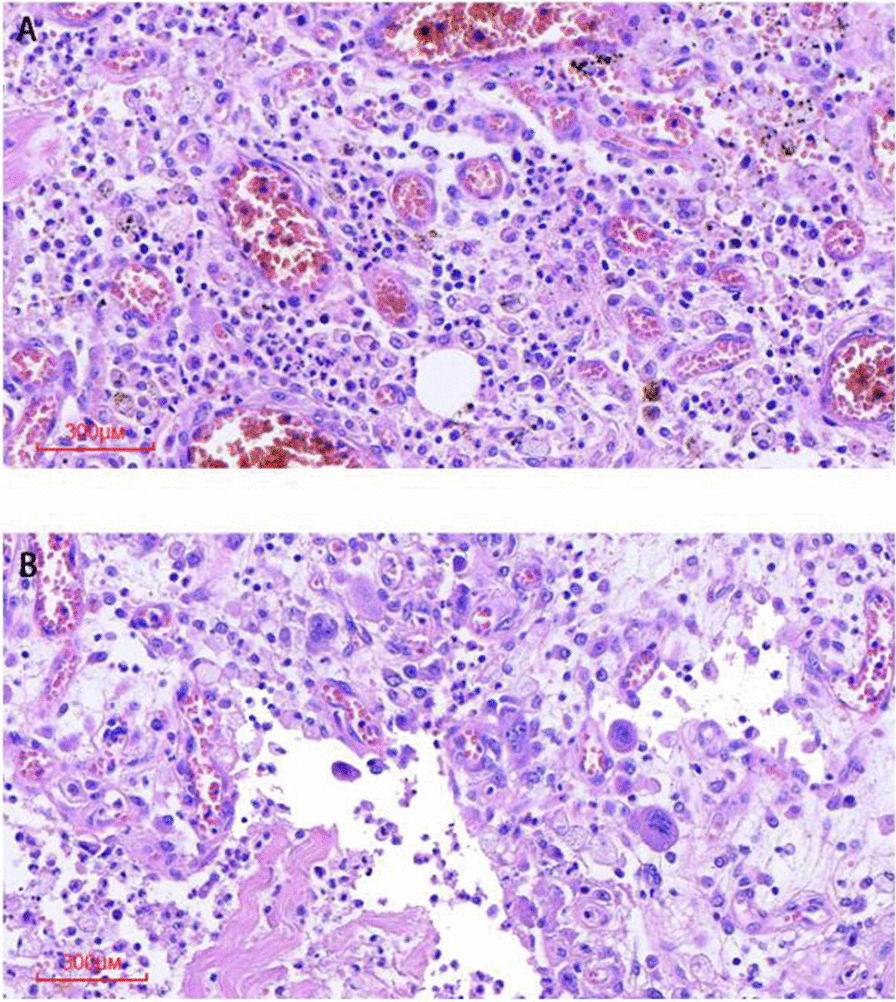
Representative pathological photomicrograph demonstrated thrombus (**A**) and abscess formation (**B**)

The patient was taken to the operating room. The neck mass adhered tightly to the surrounding tissue, and the involved segment of the left internal jugular vein was exposed by sharp dissection (Fig. 3A). The proximal part of the mass needed to be ligated first to avoid small thrombus detachment. There was a large amount of inflammation and fibrosis present in the involved area. The involved segment of the internal jugular vein and the whole neck mass were completely resected, and the distal part of the internal jugular vein was fully ligated (Fig. 3B). The pathological examination showed hemorrhagic necrosis with the proliferation of fibrous and granulation tissue and the accumulation of foam-like cells and multinuclear giant cells (Fig. 4). The patient recovered and was discharged a week after surgery. No other adverse events happened.

## Literature review

It is rare that patients with Lemierre’s syndrome require surgical intervention when antibiotics and anticoagulant therapies fail. This is the first study to summarize cases of LS requiring surgical treatment (not including abscess drainage). First, we decided to collect previous reviews and meta-analyses to obtain good knowledge of the rate of surgery in LS. Search strategy and selection criteria were as follows. A search of the literature in MEDLINE was performed through PubMed to identify relevant English language articles from 1980 to 2022. The following search terms were used: “Lemierre’s syndrome/disease and review, meta-analysis or retrospective study” and “Lemierre’s syndrome/disease and internal jugular vein”. The references of the retrieved articles were also reviewed to identify additional sources. Through reading the abstract and the full text, we found a total of six reviews and meta-analyses (Table 1) that included detailed descriptions of patients who underwent surgery (not including abscess drainage). In a retrospective review from 1998 to 2010 at a local tertiary referral hospital, 17 of the 23 patients underwent surgical treatment of the primary infection site [8]. In a 5-year systematic review, surgical procedures, such as tooth extraction, craniotomy, and ligation of the occluded vein, were performed in five patients to prevent further septic emboli [9]. A retrospective study from June 2000 to May 2016 showed that IJV ligation was performed in only one of five LS cases at the Children’s Hospital of Alabama [10]. In an 8-year Swedish nationwide retrospective study, three patients with peritonsillitis were surgically treated by tonsillectomy [11]. In the latest meta-analysis of 394 patients in 2020, only 10 patients underwent IJV ligation/excision, only 1 patient underwent ligation/excision of the thrombosed external carotid artery, 3 underwent endoscopic sinus surgery, and 11 underwent mastoidectomy [12]. In the latest systematic review, which included the most LS cases reported, surgical procedures were performed in 101 patients, and 31 patients underwent IJV ligation/embolectomy [13].

**Table 1:** Rate of surgery in LS in previous reviews or meta-analyses

Author	Type	Year	Rate of surgery	Rate of IJV ligation	Rate of pathogen not isolated
Schubert [8]	Retrospective study	2014	17/23	No description	3/23
Johannesen *et al*. [9]	Systematic review	2016	5/137	3/137	40/137
Jariwala *et al*. [10]	Retrospective study	2017	1/7	1/7	2/7
Nygren *et al*. [11]	Retrospective study	2019	3/104	No description	0/104
Gore [12]	Meta-analysis	2020	25/394	10/394	40/394
Valerio [13]	Meta-analysis	2021	101/652	31/652	69/652

## Discussion

In this study, we report a case of Lemierre’s syndrome in an elderly male caused by an acute infectious neck mass. Timely comprehensive medical and surgical treatments were given to avoid serious complications.

Internal jugular vein thrombosis is a rare and serious emergent disease that needs to be identified early in the course, as it can lead to catastrophic consequences, such as stroke or pulmonary embolism. The main pathological basis of internal jugular vein thrombosis is as follows: (1) injury of venous intima; (2) slowing down of blood flow; and (3) hypercoagulability. The common causes are as follows [14–16]: (1) facial infection, such as furuncle and carbuncle, sinusitis, otitis media, and suppurative tonsillitis; bacteria can spread through the damaged mucosa and parapharyngeal space or invade the jugular vein through the lymphatic and venous systems, leading to infectious phlebitis and bacterial embolism information; (2) long duration of internal jugular vein catheterization; (3) head and neck surgery; (4) head and neck tumor; (5) pulmonary embolism; and (6) other systemic diseases, such as polycythemia. Doctors need to take care of patients immediately when encountering such cases.

Lemierre’s syndrome can show the typical symptoms and signs of progressive infection, including sore throat, fever, or neck pain. A systematic review of Lemierre’s syndrome by Peter *et al*. found that in 84 patients, the most common first clinical presentation was a sore throat (33%), followed by a neck mass (23%) and neck pain (20%) [1]. In the current case, the patient presented with fever and neck pain at first, followed by a neck mass. The use of antibiotics before he was transferred to our hospital was ineffective. The white blood cell count, PCT level, and erythrocyte sedimentation rate (ESR) were elevated, and blood appeared in his urine. We adjusted the treatment to the combined application of broad-spectrum antibiotics, including imipenem and teicoplanin, for another 2 weeks. The patient’s body temperature returned to normal and laboratory testing showed that the patient’s infectious condition had been controlled, but the neck mass and internal jugular vein thrombosis persisted and required surgical treatment.

**Fig. 5:**
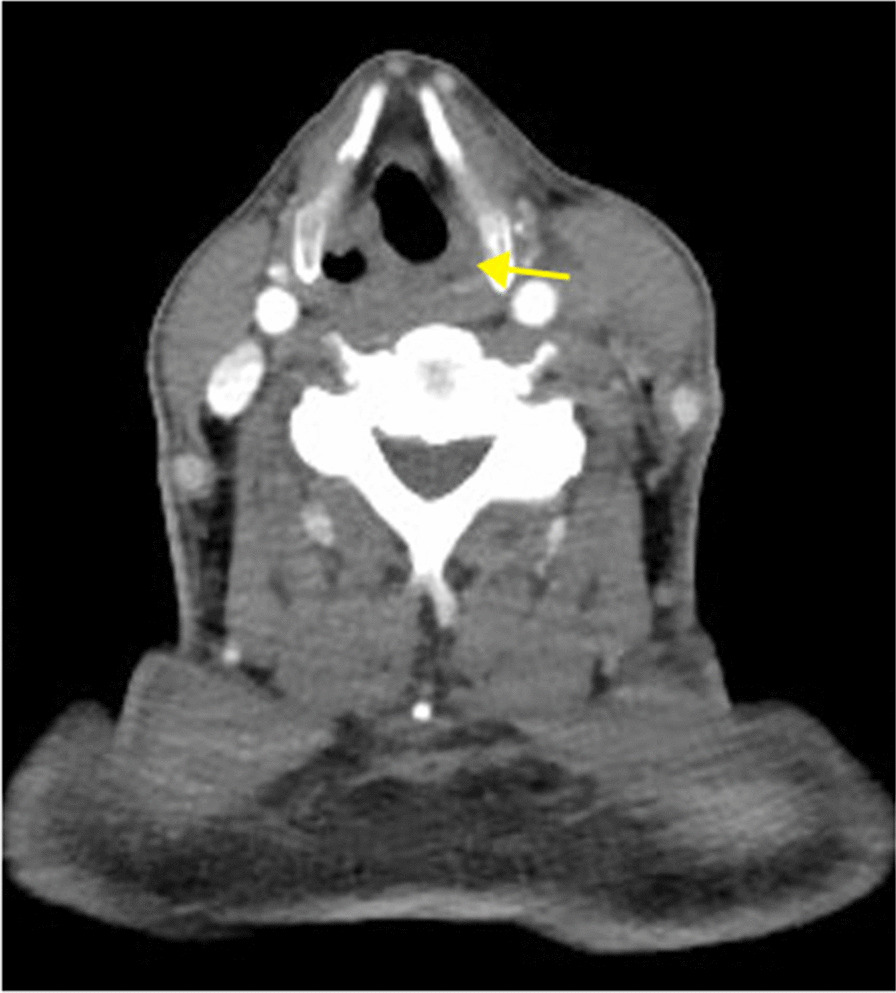
Coronal plane of computed tomography showed disappearance of left pyriform sinus surrounding by abnormal enhancement (yellow arrow)

Fusobacterium necrophorum is the main pathogen of Lemierre’s syndrome [1]. However, in this case, the culture of both blood and fluid obtained from the mass was negative for bacteria, which might be because the patient had been treated with antibiotics (mainly including ceftriaxone and metronidazole) for nearly 2 weeks before coming to our hospital. This also suggests that it is particularly important for doctors to culture blood or fluid from the mass before any use of antibiotics in these patients. In many reviews, a large proportion of the cases also did not report any microbiological agent [9, 12, 13] and thus far a clinical diagnosis of LS is still valid if the bacteria go undetected. As for the reason about how this patient got such infectious disease, considering that he had eaten mud fish before the onset of disease and CT showed that mucous of left pyriform sinus was edematous and enhanced (Fig. 5), we considered that neck infection might be secondary to the infection of mucous scratch in left pyriform sinus, and uncontrolled hyperglycemia was an important factor to cause serious infection.

The diagnosis of internal jugular vein thrombosis in Lemierre’s syndrome relies on imaging examination. Ultrasound is the first choice for the diagnosis of LS, and CT and magnetic resonance imaging (MRI) are currently implemented in general practice when necessary. Albertyn *et al*. first summarized the classic imaging features of internal jugular vein thrombosis. Ultrasonography shows the vein to be distended and nonpulsatile, with internal echoes. CT shows swelling of the adjacent soft tissues, distension of the vein with wall enhancement, and low-attenuation intraluminal filling defects. However, ultrasound has limitations and cannot display the anatomy behind the clavicle or mandible [16]. In this case, we found that although US can clearly show internal jugular vein thrombosis, knowing its boundaries and connection with the tumor still depends on CT or MRI, especially when there is an urgent need for surgery. A full assessment by preoperative imaging is of great importance. This is also consistent with the views of Charles *et al*. [17].

Priority treatment for LS includes antibiotic therapy and drainage of the infected site. Here in our hospital, considering that cephalosporins such as ceftriaxone did not control the fever and pain in this patient, imipenem and teicoplanin were used according to experience to cover wide varieties of Gram-negative and Gram-positive bacteria, including both anaerobic and aerobic bacteria. In addition, teicoplanin has lower side effects than vancomycin [18]. Rarely, other surgical procedures, such as ligation of the occluded vein, craniotomy, and tooth extraction, are performed. Antithrombotic therapy, including novel oral anticoagulants (DOACs), is also recommended depending on the individual’s condition. However, it remains controversial whether anticoagulation or antithrombotics are effective in Lemierre’s syndrome. Some scholars think that thrombosis is due to the infection process and can be resolved when the infection has been controlled [19]. In this case, we did not immediately apply anticoagulant or thrombolytic therapies considering that the APTT of this patient was significantly prolonged at the time he came to our hospital and the consumption of platelets was relatively high; emergency anticoagulant therapy may have increased his risk of bleeding. To date, there have been no sufficient clinical studies and no sufficient evidence suggesting the necessity for anticoagulant therapy in Lemierre’s syndrome [20]. Previous studies have also reported the occurrence of extensive suppurative thrombophlebitis of the bilateral IJV and superior vena cava in patients with Lemierre’s syndrome despite the use of antibiotics and anticoagulant therapy; adjunctive catheter-directed thrombolysis and superior vena cava stenting were performed to help these patients completely recover [21]. Anticoagulation therapy has not been shown to reduce the complications of Lemierre’s syndrome, such as sepsis [17]. Meanwhile, Johannesen *et al*. did not find that anticoagulation therapy decreased the mortality rate or course of the disease or reduced the duration of antibiotic use [9]. However, anticoagulation therapy is recommended in patients with a poor clinical response despite antibiotic therapy and with a high risk of intracranial thrombosis or recurrent thrombophlebitis [22–24]. In this case, the cause of internal jugular vein thrombosis was largely infection, so surgical treatment was the best choice when antibiotics could not completely cure the infection and thrombosis. Through previous retrospective studies, systematic reviews and meta-analyses obtained by database searches, we summarized the following points regarding surgical intervention in Lemierre’s syndrome:

1. When patients do not respond to conservative medical therapy and continue to show extensive septic thrombosis or uncontrolled severe sepsis, surgical treatments need to be considered.
2. Abscess drainage is the most common and convenient surgical treatment for abscesses upon formation.
3. Surgical treatment of the primary infection site is effective for controlling the spread of infection and sepsis.
4. IJV ligation or excision is suitable for patients with persistent septic embolization after treatment with antibiotics and anticoagulants.
5. IJV ligation or excision is also appropriate to avoid thrombus detachment when anticoagulation therapy or catheter-directed thrombolysis is ineffective.

## Conclusion

Lemierre’s syndrome is an extremely rare disease, but the fatality rate can reach 15%, even with escalating antibiotic therapy [21]**.** Therefore, early diagnosis is particularly important, and the timely institution of appropriate antibiotics is the standard treatment. Surgical intervention may be the only effective option for controlling the source of infection or when conservative medical treatment fails.

## Data Availability

Available from corresponding author on reasonable request.

## Abbreviations

IJV: Internal jugular vein
ENT: Ear, nose, and throat
LS: Lemierre’s syndrome
WBC: White blood cell
PLT: Platelet
CRP: C-reactive protein
PCT: Procalcitonin
BP: Blood pressure
CT: Computed tomography
MRI: Magnetic resonance imaging
